# Engineering the Healing Process: Advanced In Vitro Wound Models and Technologies

**DOI:** 10.3390/biomedicines14040754

**Published:** 2026-03-26

**Authors:** Filippo Renò, Mario Migliario, Maurizio Sabbatini

**Affiliations:** 1 Health Sciences Department, Università of Milan, Via A. di Rudini n. 8, 20142 Milan, Italy; filippo.reno@unimi.it; 2 Traslational Medicine Department, Università del Piemonte Orientale, Via Solaroli n. 17, 28100 Novara, Italy; mario.migliario@med.uniupo.it; 3 Sciences and Innovative Technology Department, Università del Piemonte Orientale, Viale T. Michel 11, 15121 Alessandria, Italy

**Keywords:** 3D bioprinting, organ-on-chip, microfluidic, organoid, skin engineering, iPSC-based models

## Abstract

Advances in regenerative medicine increasingly rely on human-relevant in vitro systems to model the multistage process of wound healing. However, the translation of research into effective therapies remains limited by the inability of traditional 2D cultures and animal models to faithfully replicate the structural and biochemical complexity of human skin. While existing reviews often focus on the structural composition of static skin equivalents, this review addresses a critical knowledge gap: the need for dynamic, time-dependent methodologies that can capture the spatiotemporal evolution of healing, from inflammation to remodeling, in both physiological and pathological conditions. To this end, we critically evaluate next-generation platforms, including 3D bioprinting, organ-on-chip systems, organoids, and iPSC-based models, highlighting their comparative advantages and technical hurdles like vascularization and scalability. The unique contribution of this work lies in providing a forward-looking framework that advocates for the convergence of bioengineering and computational modeling to move beyond “steady-state” snapshots toward predictive, high-resolution dynamic models. We conclude that the future of wound healing research depends on integrating vascular and immune components within these platforms to achieve truly human-relevant, personalized diagnostic and therapeutic tools.

## 1. Introduction

Understanding the complex and dynamic process of wound healing remains a central goal in regenerative medicine. This multifactorial biological event involves a tightly regulated sequence of hemostasis, inflammation, proliferation, and remodeling [1]. While traditional two-dimensional (2D) cell cultures and animal models have provided insights into these processes, they fail to fully recapitulate the structural and immunological complexity of human skin, often leading to limited translational success [2,3]. These limitations have spurred the development of next-generation in vitro models that aim to bridge the gap between reductionist systems and human physiology [4]. Advanced three-dimensional, tissue-engineered constructs, such as organotypic skin equivalents and microphysiological systems (“skin-on-a-chip”), now allow researchers to mimic the native architecture and multicellular interactions of human skin [5]. By integrating key components such as ECM scaffolds, vascular networks, and immune cells, these models provide a more accurate platform for studying wound healing dynamics, drug responses, and biomaterial biocompatibility. Moreover, the incorporation of human-derived matrices, such as the human amniotic membrane (hAM), offers new opportunities to explore the regenerative potential of naturally bioactive substrates within a controlled environment [6], accelerating the translation of regenerative therapies from bench to bedside [7].

Furthermore, in recent years, the 3Rs principle of Russell and Burch [8] has received renewed attention [9,10]. This principle is a foundational ethical framework guiding the use of animals in scientific research: replacement encourages the use of alternative methods, such as in vitro models, computer simulations, or human-based studies, whenever possible; reduction aims to minimize the number of animals used while still obtaining reliable and valid results, often through improved study design and statistical planning; and refinement focuses on modifying procedures and husbandry practices to reduce pain, suffering, and distress, thereby improving animal welfare. Together, the 3Rs promote responsible research by balancing scientific advancement with ethical obligations toward animals. These evolving models of wound healing hold promise for reducing dependence on animal testing, reinforcing the adoption of the 3Rs principle.

**Figure 1:**
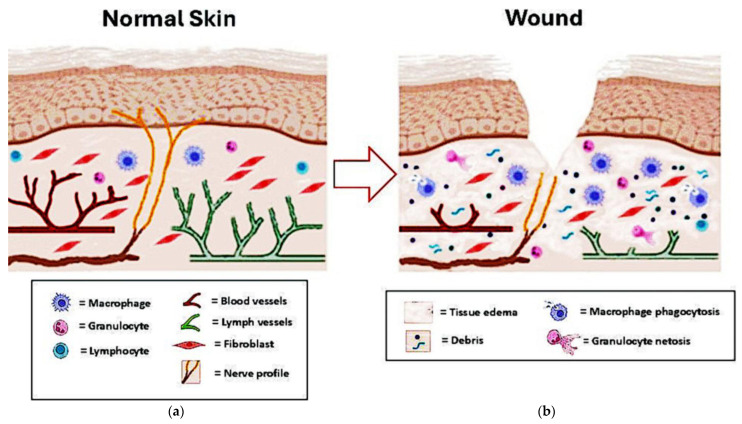
(**a**) Skin schematic structure; (**b**) early modifications induced by wounding (created in BioRender. Sabbatini, M. (2026) https://BioRender.com/0yhwwyg, accessed on 20 March 2026).

Despite the emergence of advanced three-dimensional (3D) tissue-engineered constructs and microphysiological systems, a significant knowledge gap persists, most current in vitro evaluations rely on “steady-state” or static analysis, which focuses on the structural equilibrium of tissues under homeostatic conditions. In contrast, wound healing is a spatiotemporal process that requires dynamic, time-dependent methodologies to capture the evolution of cellular and molecular signals across different healing phases (Figure 1).

Therefore, it becomes necessary to adopt dynamic, time-dependent methodologies capable of highlighting the ongoing dynamics and describing the evolution of the healing process, in both physiological and pathological conditions, allowing for a more realistic understanding of the underlying biological mechanisms and a more accurate assessment of therapeutic interventions, even in models that could be more personalized using patient-derived cells.

The objective of this review is to critically evaluate next-generation human-relevant platforms, including 3D bioprinting, organ-on-chip systems, organoids, and iPSC-based models, specifically through the lens of their ability to move beyond static snapshots toward dynamic, predictive modeling of the wound healing process. By identifying current technical limitations in vascularization and standardization, this work provides a framework for developing ethical and clinically translatable models capable of simulating both physiological and pathological healing dynamics.

The present review aims to discuss the various methodological approaches available for steady-state analysis of the skin, as well as methods for dynamically assessing the wound healing process.

## 2. Old Style Models: 2D Cell Cultures and Animal Models

In 2D monolayer cultures, cells grow on rigid plastic surfaces under static conditions, lacking the three-dimensional (3D) mechanical and biochemical context of native tissue. These systems cannot faithfully reproduce gradients of oxygen, nutrients, or signaling molecules, nor do they replicate the spatial organization of skin layers. Moreover, cell morphology, polarity, and differentiation pathways are often altered under these artificial conditions, influencing gene expression profiles and functional behavior [11]. As a result, 2D systems offer an oversimplified representation of wound healing, capturing only isolated cellular events rather than a collective tissue response [2]. Furthermore, conventional models poorly replicate the complex in vivo microenvironment, particularly the dynamic biomechanical forces (tension, compression, and shear) that strongly influence healing. Traditional mechanical stimulation is difficult to standardize and control, reducing the predictive value and translational relevance of these models [12].

Animal models, although essential for studying systemic responses and wound closure kinetics, exhibit significant interspecies variability in skin structure, immune response, and healing dynamics [13]. For example, rodents heal predominantly through wound contraction, whereas humans rely more on re-epithelialization and granulation tissue formation [14]. Such fundamental differences limit the translatability of animal data to human clinical outcomes. Ethical concerns, cost, and reproducibility further constrain their utility, highlighting the need for more physiologically representative alternatives [15].

While in vitro models are rapidly advancing, they are currently viewed as complementary to, rather than a full replacement for animal studies. In vivo models provide systemic integration, encompassing the nervous, endocrine, and circulatory systems, that remain beyond the reach of current benchtop technologies. Animal testing remains a mandatory step in the regulatory pipeline for the safety assessment of new clinical therapies. However, high-fidelity in vitro systems serve to refine animal use by providing human-specific mechanistic data and filtering out ineffective compounds early in the development process [16,17].

### Methods for In Vitro Wound Induction

The fidelity of an in vitro model is intrinsically linked to the method used to simulate injury. Traditional ‘scratch assays’ in 2D monolayers fail to capture the depth-dependent healing seen in vivo. In 3D tissue-engineered models, mechanical induction via biopsy punching or micro-scalpels better mimics surgical or excisional wounds. Alternatively, laser-induced thermal injury (e.g., CO_2_ lasers) allows for the study of burn-specific dynamics, including the formation of a necrotic zone. Recent microfluidic innovations utilize localized chemical flows, such as trypsin or focused oxidative stress, to create ‘virtual’ wounds without compromising the integrity of the extracellular matrix (ECM) [18,19].

## 3. 3D Tissue-Engineered Skin Equivalents

Among the most promising innovations are 3D tissue-engineered skin equivalents [20], which integrate dermal and epidermal layers composed of primary human keratinocytes and fibroblasts within biomimetic scaffolds [21,22]. Hydrogels such as collagen, fibrin, gelatin methacrylate (GelMA), and decellularized extracellular matrix (dECM) provide a supportive niche that mimics the native microenvironment [23]. These models enable the study of cell–cell and cell–matrix interactions under controlled conditions, supporting the real-time evaluation of processes such as re-epithelialization, angiogenesis, and ECM remodeling [4,24]. Consequently, they serve as a valuable starting point for wound-healing models (Figure 1).

These models bridge the gap between conventional in vitro systems and clinical applications, providing physiologically relevant platforms for regenerative medicine and drug screening [25,26]. For example, patient-derived keratinocytes and fibroblasts can be used to generate autologous skin equivalents, enabling personalized studies of genetic skin disorders, wound healing deficits, or responses to therapeutic interventions [25] (Table 1).

### 3.1. Mechanism and Development Strategy

The core mechanism of 3D tissue-engineered skin equivalents (TESEs) revolves around the spatial recapitulation of the human skin’s hierarchical architecture, moving beyond the limitations of traditional 2D monolayers [21,23]. The strategy for developing these models involves a staged bio-fabrication process such as dermal reconstitution, where fibroblasts are embedded within a supportive matrix, frequently a hydrogel, to create a “dermal equivalent.” This allows for natural cell–matrix interactions and provides the mechanical tension necessary for tissue stability [27]. A critical strategic element is the exposure of these cells to an air-liquid interface. This process triggers terminal differentiation, leading to the formation of a functional stratum corneum, which mimics the human skin’s physical barrier [28].

### 3.2. Evaluation of Hydrogel Formulations: Mechanism and Advantages

The use of 3D skin equivalents is particularly advantageous for the evaluation of hydrogel-based formulations (e.g., collagen, fibrin, or synthetic polymer scaffolds) as Hydrogels serve as an analogue to the natural extracellular matrix (ECM). In a 3D system, we can evaluate how the physical properties of a hydrogel, such as its stiffness and porosity, directly influence cellular behavior, including fibroblast migration and myofibroblast differentiation, which are key drivers of the remodeling phase [29,30]. Unlike 2D models, 3D TESEs allow for the observation of re-epithelialization in a physiologically relevant context. Researchers can monitor how a hydrogel formulation supports or accelerates the migration of keratinocytes across a wounded area, providing a more accurate predictive tool for wound closure rates [22,31]. These systems provide a standardized platform to test “bio-instructive” hydrogels loaded with growth factors or drugs. Because these models utilize human-derived cells, they offer a higher predictive value for human clinical outcomes compared to animal models, which often heal through contraction rather than re-epithelialization]. By integrating these complex formulations into a 3D architecture, the system transitions from a simple scaffold to a dynamic ecosystem, allowing for a comprehensive analysis of how a hydrogel guides the biological stages of the healing process (Table 1).

### 3.3. Biomaterials and Mechanical Cues in Wound Modeling

Biomaterial selection dictates the structural and functional outcome of skin equivalents. While natural polymers (e.g., collagen, fibrin, hyaluronic acid) provide essential cell-recognition sites, synthetic polymers like polyethylene glycol (PEG) or polycaprolactone (PCL) offer superior control over mechanical properties. The ‘stiffness’ of the scaffold is a key driver of cell fate; for instance, matrices with high Young’s moduli can induce myofibroblast differentiation, leading to a fibrotic phenotype characteristic of hypertrophic scarring [32]. Furthermore, decellularized extracellular matrix (dECM) bioinks are increasingly used to provide a niche that preserves the native tissue’s biochemical complexity [33] (Table 1).

### 3.4. The Functional Role of Additional Cell Types

Recent studies have incorporated additional cell types, such as melanocytes, endothelial cells, macrophages, and sensory neurons, creating multi-layered organotypic constructs that better reflect the physiological heterogeneity of human skin [34,35,36,37]. Moreover, dynamic culture systems such as bioreactors and perfusion chambers allow for continuous nutrient exchange and mechanical stimulation, promoting tissue maturation and long-term viability [38,39]. To further bridge the gap between in vitro results and clinical outcomes, the inclusion of specialized cell types is essential. Rather than adding simple complexity, each cell type introduces a critical functional attribute for wound studies. Endothelial cells integration allows for the study of angiogenesis and vascular network formation, which is vital for nutrient delivery to the healing tissue [40]. Macrophages provide an immunological component, allowing researchers to evaluate how a treatment helps resolve chronic inflammation, a primary barrier in non-healing wounds like diabetic ulcers [40,41]. The skin is also a sensory organ; including neurons enables the study of neurogenic inflammation and the evaluation of how topical treatments might influence pain signaling or nerve regeneration [42]. Melanocytes are crucial for observing the restoration of the pigmentary barrier and understanding post-inflammatory skin changes following the healing process [34,42].

By adopting this multi-cellular strategy, 3D skin equivalents evolve from static structural scaffolds into dynamic, “organ-on-a-chip” style ecosystems. This provides a robust platform for the predictive screening of new therapies, ultimately enhancing the safety and efficacy of clinical translations in wound care [43].

### 3.5. Clinical Success and Regulatory Challenges

While advanced in vitro platforms offer superior physiological relevance compared to traditional 2D cultures and animal models, their transition from the laboratory to clinical and industrial use is governed by a distinct set of regulatory and translational hurdles.

These models have seen significant success as autologous grafts for treating genetic skin disorders and chronic wound deficits. They are currently a cornerstone in toxicology testing and for studying patient-specific responses to therapeutic interventions.

However, standardizing these constructs remains a major hurdle due to the inherent variability of primary human cell sources. Ensuring long-term stability and full immunocompatibility for host integration after transplantation are critical requirements for broader regulatory approval.

### 3.6. Advantages and Limitations

**Figure 2:**
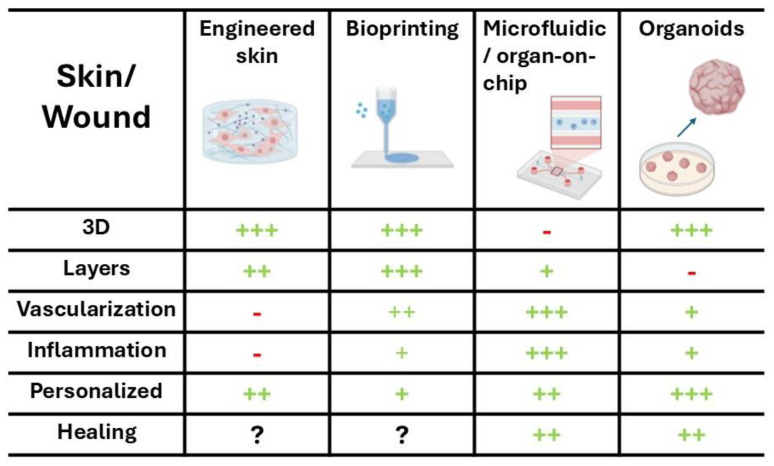
Reproducibility of the structural and functional characteristics of the skin and wound by the various technologies used (created in BioRender. Sabbatini, M. (2026) https://BioRender.com/hf94q57, accessed on 20 March 2026).

Looking forward, the convergence of 3D bioprinting, stem cell technology, and advanced biomaterials promises to create highly reproducible, physiologically relevant skin constructs. These can serve as powerful tools for regenerative medicine, toxicology testing, and the study of complex skin pathologies. Such innovations are poised to bridge the gap between conventional in vitro models and clinical applications, ultimately paving the way for more predictive and personalized approaches to skin biology and therapy (Figure 2).

Despite these advances, several challenges remain [44]. Reproducing complex features, such as hair follicles, sweat glands, sebaceous units, and the intricate vascular and neural networks of native skin is still limited, restricting the capacity of current models to fully emulate in vivo physiology [45]. Additionally, long-term culture stability, immunocompatibility, and integration with host tissue after transplantation are critical hurdles for clinical translation [46,47]. Furthermore, the methodology for effectively inducing a “wound” and accurately monitoring its subsequent repair processes remains a significant technical challenge in these models (Table 1).

**Table 1:** Key Biomaterials Used in Skin Engineering.

Material	Crosslinking	Advantages	Limitations	Applications
Collagen [48]	Enzymatic/pH	Biocompatibility	Low mechanics	Dermal scaffolds
GelMA [49]	Photo-crosslink	Tunable stiffness	UV toxicity risk	Bioinks; microchips
Fibrin [50]	Enzymatic	Pro-angiogenic	High shrinkage	Acute wound clots
dECM [51]	Chemical/thermal	Native factors	Standardization	Specific modeling
Hyaluronic Acid [52]	Chemical	Hydration	Poor cell adhesion	Chronic wounds
Alginate [53]	Ionic (Ca^2+^)	Ease of use	Low bioactivity	Bioprinting
Synthetic (PEG) [54]	Chemical/photo	Reproducibility	Bio-inert	Mechanobiology

GelMA: gelatin methacryloyl; dECM: decellularized extracellular matrix; PEG: poly(ethylene glycol).

## 4. Critical Perspective for In Vitro Platforms

### 4.1. Challenges in Cell Sourcing and Phenotypic Stability

A critical consideration in the development of these models is the selection of the cell source, which carries inherent biological trade-offs. While the use of primary human cells is ideal for maintaining physiological relevance, their application is often constrained by limited expansion potential and high donor-to-donor variability, particularly when attempting precise cell deposition in complex architectures [55]. Furthermore, it is technically impossible to use exclusively primary cells for all components; for example, primary endothelial cells required for vascularization studies are difficult to reproduce and notoriously lose their specialized phenotype and functional maturity in long-term culture [56]. Consequently, many researchers utilize immortalized or surrogate cell lines to ensure experimental reproducibility and scalability in multi-layered organotypic constructs.

However, the physiology of these surrogate lines often differs markedly from typical primary cultures, exhibiting altered metabolic profiles and shifted signaling pathways [57]. Beyond the cell source itself, the behavior of cells in vitro may diverge significantly from in vivo responses. This is often due to partial dedifferentiation upon transition to a 2D or 3D culture environment, which alters fundamental signaling pathways [58]; altered mechanotransduction in the absence of native niche forces and the dynamic extracellular matrix [59]; or the loss of systemic tissue-specific signals, including the complex interplay of immune and mechanical forces [25].

These metabolic and functional shifts can result in cellular responses that do not fully capture the complexity of human wound repair in vivo.

### 4.2. Immune Competence and Inflammation Modeling

Wound healing is critically governed by a highly coordinated and temporally regulated immune response that extends far beyond the mere presence of inflammatory cells. During the early inflammatory phase, neutrophils are rapidly recruited to the wound site through chemokine gradients, where they exert antimicrobial functions not only via phagocytosis and degranulation but also through the formation of neutrophil extracellular traps (NETosis), a process that, while protective, may contribute to tissue damage and chronic inflammation if dysregulated [12]. Monocytes subsequently infiltrate the wound and differentiate into macrophages, whose phenotypic plasticity plays a pivotal role in determining healing outcomes. Classically activated M1 macrophages dominate the early phase, producing pro-inflammatory cytokines such as TNF-α and IL-1β to clear debris and pathogens, whereas a timely transition toward alternatively activated M2 phenotypes supports resolution of inflammation, angiogenesis, extracellular matrix deposition, and tissue remodeling [12,60]. Impairment of this M1-to-M2 switch is a hallmark of chronic wounds, including diabetic ulcers [61,62]. In parallel, adaptive immune components contribute to wound chronicity and regulation: distinct T-cell subsets modulate macrophage polarization and fibroblast activity through cytokine secretion, while B cells may influence healing through antibody production and regulatory signaling [60,63].

Importantly, immune–epithelial cross-talk represents a central regulatory axis in tissue repair. Keratinocytes and fibroblasts actively participate in immune modulation by secreting cytokines and chemokines that shape leukocyte recruitment and activation, establishing dynamic paracrine feedback loops that coordinate re-epithelialization and matrix remodeling [12,63]. In addition, the cutaneous microbiome exerts a profound influence on the inflammatory milieu; commensal bacteria may promote balanced immune activation and barrier restoration, whereas pathogenic colonization and biofilm formation sustain persistent inflammation and delay healing [64].

The lack of immunological depth in current platforms remains a significant hurdle, as traditional systems often overlook the critical transitions and specialized roles of immune cell subsets. For instance, capturing the spatiotemporal switch in macrophage polarization, from the pro-inflammatory M1 phenotype to the pro-repair M2 phenotype, is essential for modeling the transition from inflammation to proliferation. Furthermore, the early innate response, characterized by neutrophil recruitment and the formation of Neutrophil Extracellular Traps (NETosis), provides the foundational signals for subsequent repair phases but is rarely integrated into static models. In chronic scenarios, such as diabetic ulcers, the lymphocyte involvement (specifically T-cell and B-cell dysfunction) significantly impairs healing, highlighting the need for models that can simulate these specific pathological immune states. This complexity is further compounded by immune-epithelial cross-talk, where cytokine gradients and paracrine loops between immune cells and keratinocytes dictate the regenerative trajectory. Finally, integrating the microbiome is vital, as the presence of commensal or pathogenic bacteria can drastically alter the inflammatory profile and the overall speed of wound closure.

### 4.3. Skin Innervation

The skin is one of the most densely innervated organs, containing extensive networks of sensory nerve fibers that interact with keratinocytes, fibroblasts, immune cells, and vascular structures. This neuro-cutaneous system plays a crucial regulatory role during wound healing through the release of neuropeptides such as substance P (SP), calcitonin gene-related peptide (CGRP), and vasoactive intestinal peptide (VIP), which modulate inflammation, angiogenesis, and cellular proliferation [65]. Sensory neuron-derived mediators can stimulate keratinocyte migration, fibroblast activation, and extracellular matrix remodeling, thereby accelerating re-epithelialization and tissue regeneration. For example, in vitro studies using tissue-engineered skin models co-cultured with sensory neurons demonstrated significantly faster wound closure due to neuropeptide signaling, particularly SP acting through the neurokinin-1 receptor on keratinocytes [42].

Despite this well-recognized physiological role, neural components remain largely absent from most advanced in vitro wound models, which typically focus on epithelial, fibroblast, immune, and endothelial interactions. Emerging approaches aim to address this gap by incorporating sensory neuron co-cultures, microfluidic neurovascular platforms, and disease-relevant systems such as diabetic neuropathy models, where impaired innervation and reduced neuropeptide signaling are associated with delayed healing [66]. Integrating neural elements into engineered wound models is therefore essential to more accurately reproduce the complexity of human skin repair and to study neurogenic mechanisms underlying chronic wounds.

## 5. Advanced Strategies for In Vitro Vascularization and Perfusion

Vascularization remains one of the most significant challenges in skin engineering, as the lack of a functional nutrient delivery system limits the thickness and viability of 3D constructs. To address this, current research has shifted from monocultures to co-culture systems with endothelial cells (ECs), often using human umbilical vein endothelial cells (HUVECs) or microvascular ECs integrated within a dermal matrix. These cells spontaneously organize into capillary-like structures when provided with appropriate pro-angiogenic cues, such as VEGF-enriched media or co-culture with supportive pericytes and fibroblasts [67].

Beyond spontaneous organization, pre-vascularization techniques utilize biofabrication tools like 3D bioprinting and sacrificial molding to create predefined hollow channels. These channels can be lined with endothelial cells to form a primitive endothelium, significantly accelerating the integration of the graft with the host’s vasculature upon potential transplantation [68]. However, static cultures fail to replicate the hemodynamic forces crucial for vascular maturation. Consequently, perfusion systems and microfluidic bioreactors have been developed to introduce interstitial flow and shear stress, which promote the alignment of ECs and enhance the barrier function of the newly formed vessels [68].

Despite these advances, several limitations persist. Most current models lack a hierarchical vascular architecture (arterioles–capillaries–venules), and the long-term stability of in vitro networks remains tenuous. Furthermore, replicating the complex interplay between the immune system and the vascular endothelium during the inflammatory phase of wound healing remains a frontier that requires more sophisticated multi-organ-on-chip integration.

## 6. Bioprinting Technologies

3D bioprinting has revolutionized the fabrication of tissue models by enabling precise spatial deposition of cells and biomaterials according to predefined digital architectures [18,20,69]. This technique allows for the recreation of skin geometries, gradients of stiffness, and heterogeneous tissue compositions that mirror the complex topography of skin. Bioprinted constructs can be designed to include vascular networks or gradients of growth factors such as VEGF and PDGF to simulate angiogenesis and tissue regeneration [70,71]. Furthermore, bioprinting provides a powerful tool for high-throughput screening of novel biomaterials, growth factors, and pharmacological agents in a reproducible manner [72,73].

Traditional engineered skin models include a dermis with fibroblasts embedded in a collagen gel and an overlying keratinocyte layer, but more advanced models now incorporate endothelial cells, skin appendages, vascular networks, and more complex ECM. 3D systems promote more physiological cellular behavior compared to 2D cultures: keratinocyte migration, collagen deposition by fibroblasts, interactions with biomimetic scaffolds, and matrix contraction/remodeling can be monitored [4] (Table 2).

### 6.1. Clinical Success and Regulatory Challenges

Bioprinting has succeeded in recreating complex skin geometries and heterogeneous compositions, such as vascular networks and growth factor gradients, which are essential for simulating human-like angiogenesis. It has become a powerful high-throughput tool for screening novel pharmacological agents.

The high complexity of these models often leads to increased costs and reduced reproducibility across different laboratories. Standardizing the precise spatial deposition of cells and the bio-ink parameters is necessary to meet the rigorous quality assurance standards required for clinical-grade production (Table 2).

### 6.2. Advantages and Limitations

In a 3D skin model, it is possible to simulate the re-epithelialization phase by observing keratinocyte migration across the dermal–epidermal interface, study new capillary formation induced by endothelial cells, and modulate the ECM to replicate the remodeling phase. These models also allow testing “pro-regenerative” biomaterials or growth factors in a context closer to human tissue than 2D cultures [74,75] (Figure 2).

While the structural layering of these models is increasingly sophisticated, limitations persist regarding their functional dynamics. Specifically, the lack of a pressurized vascular network prevents the simulation of the biomechanical signaling and leukocyte extravasation that characterize the early inflammatory phase. This absence truncates the ‘biological crosstalk’ required to initiate the cellular signaling pathways that move the wound from injury toward active repair [40] (Table 2).

## 7. Microfluidic and Organ-on-Chip Systems

Another transformative innovation is the emergence of microfluidic organ-on-chip platforms. These systems recreate the dynamic biochemical and biomechanical features of the wound milieu by integrating perfusable channels that mimic blood vessels and interstitial flow [76,77]. Such chips reproduce oxygen gradients, nutrient flows, shear stress, and complex cell–cell interactions that are difficult to simulate in static 2D or simple 3D models.

The integration of microfluidics represents a pivotal shift from the ‘static/steady-state’ snapshots of traditional 3D models toward the ‘dynamic, time-dependent methodologies’ necessary to capture the fluid nature of repair. By implementing precise flow control, these platforms do not merely house cells; they replicate the temporal evolution of the wound bed. For instance, the ability to transition from a pro-inflammatory flow state to a pro-regenerative perfusion profile allows researchers to observe the healing process as a continuous trajectory rather than a fixed endpoint. This dynamic capability is crucial for studying the recruitment of circulating immune cells and the subsequent remodeling phase, which are intrinsically time-dependent processes that static models fail to replicate [40,78].

In recent years, the development and application of organ-on-chip (OoC) technology have accelerated translational research, enabling more efficient preclinical-to-clinical pipelines and reducing reliance on animal models [79]. Their use has expanded across biomedical research, the pharmaceutical industry, and regenerative medicine, highlighting their role not only as investigative tools but also as platforms for therapeutic innovation [80]. As technological advances continue, organs-on-chips are poised to further enhance precision medicine [81], uncover novel disease mechanisms, and drive the development of patient-specific therapeutic strategies, representing a paradigm shift in biomedical research and clinical translation.

In wound healing research, one model generated wounds of varying widths within a chip using a trypsin flow, allowing for the study of wound width and shear stress effects on healing rates [76]. Similarly, wound healing analysis has expanded following a recent study describing enhanced closure in diabetic keratinocytes using a microfluidic bioelectronic platform, where direct current stimulation created a directional electric field [82]. By introducing immune components—such as macrophages, neutrophils, and lymphocytes—and even elements of the skin microbiome, these chips enable the modeling of inflammatory responses and oxygen gradients often lost in static cultures [7,83].

Microfluidic models also facilitate real-time imaging and high-content analysis, allowing for the quantitative assessment of ECM deposition, cell migration, and cytokine release [84]. Advances in integrated biosensors now permit the continuous monitoring of pH, oxygen tension, and reactive oxygen species (ROS) within the chip, providing unprecedented temporal resolution for studying wound physiology [85]. Moreover, coupling microfluidic wound models with automated imaging pipelines and machine-learning-based pattern recognition has accelerated the discovery of subtle healing phenotypes and drug-response signatures [86].

The scalability of these platforms supports parallel experimentation for drug discovery and toxicity screening [87,88], further reducing reliance on animal testing [89]. As pharmaceutical and biomaterials companies adopt these technologies, wound-on-chip systems are increasingly recognized as a cornerstone for the preclinical evaluation of pro-regenerative therapies, engineered scaffolds, and bioactive molecules [77,90].

Ultimately, these advances position microfluidic platforms as essential tools for bridging the translational gap between laboratory innovation and clinically effective wound-healing strategies.

**Figure 3:**
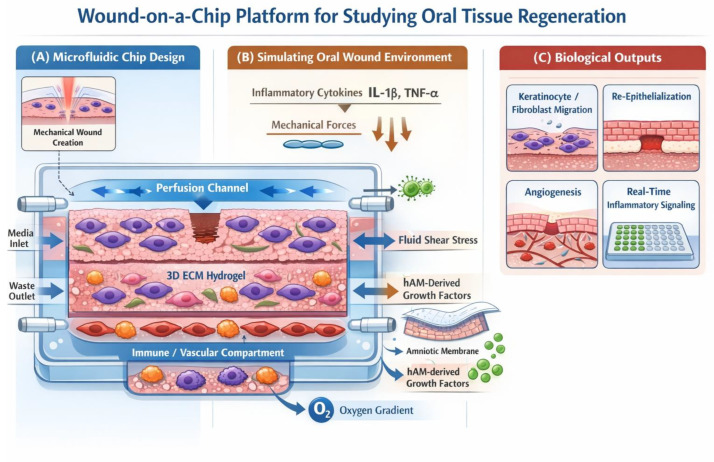
(**A**) Microfluidic design: a 3D platform with perfusion channels and hydrogel to simulate oral tissue structure and vascularization; (**B**) wound environment: simulation of trauma through mechanical stimuli, oxygen gradients, and inflammatory cytokine release; (**C**) biological outputs: real-time monitoring of healing, including cell migration, re-epithelialization, angiogenesis, and biochemical signaling (image created by “Google Gemini 3 Flash- free version” AI).

While still an emerging field, wound-on-a-chip technologies represent the frontier of precision modeling by integrating microfluidics with 3D tissue engineering. The strategic advantage of these systems lies in their ability to control the spatiotemporal microenvironment, allowing for the simulation of dynamic processes that are otherwise “invisible” in static 3D models [21,40] (Figure 3).

### 7.1. Wound-on-Chip Relevant Studies

Although wound-on-a-chip platforms are still in the early stages of development, several pioneering studies have demonstrated their potential for modeling the dynamic processes involved in tissue repair. For instance, microfluidic wound models have been developed to recreate controlled epithelial injuries within perfused channels, allowing precise investigation of keratinocyte migration and wound closure kinetics under defined shear stress and biochemical gradients. One representative example is the microfluidic scratch-wound model described by Murrel and colleagues [91], where epithelial monolayers were cultured inside a perfused microchannel and a reproducible wound was generated through localized trypsin exposure, enabling quantitative assessment of cell migration and re-epithelialization dynamics. Similarly, Zhao [92] developed a bioelectronic microfluidic platform capable of generating controlled electric fields across wounded keratinocyte layers, demonstrating that electrical stimulation significantly accelerates wound closure, particularly in diabetic cell models.

More recently, advanced skin-on-chip platforms have begun incorporating multiple cell types, including fibroblasts, endothelial cells, and immune components, within perfused microfluidic environments to better reproduce the inflammatory and vascular phases of wound healing [see Section 3.4 of the present work]. These systems allow real-time imaging, controlled delivery of growth factors or biomaterials, and continuous monitoring of cellular responses under physiologically relevant flow conditions. Although still limited by issues related to structural complexity and long-term stability, these early implementations demonstrate the feasibility of integrating controlled injury generation with microphysiological environments, providing a promising framework for future wound-on-chip technologies.

Several representative studies demonstrating the application of microfluidic and organ-on-chip technologies for investigating wound healing and tissue regeneration are summarized in Table 3.

### 7.2. Clinical Success and Regulatory Challenges

Wound-on-chip systems have successfully accelerated the discovery of subtle healing phenotypes and drug-response signatures. They have been adopted by pharmaceutical companies as cornerstone technologies for the preclinical evaluation of pro-regenerative therapies.

Despite their potential, these technologies are still in their early stages. Key regulatory barriers include the need for validated standardized protocols to ensure that findings from these dynamic systems can reliably replace data currently derived from animal models.

### 7.3. Advantages and Limitations

These systems allow high-throughput, real-time monitoring, precise manipulation of microenvironments and stimuli (mechanical, electrical, chemical), and reduce ethical and financial burdens compared to animal models. Challenges remain, such as 3D structure and an extended layering, long-term studies, standardization between labs, and scalability for high-throughput drug/material screening (Figure 2).

## 8. Skin Organoids

Organoids have increasingly been recognized as a transformative platform for advancing the understanding of complex biological mechanisms. As highlighted in the broader organoid literature, these three-dimensional systems recapitulate key structural and functional elements of human tissues, thereby providing a physiologically relevant environment for studying complex regenerative processes [96]. In the context of cutaneous repair, skin organoids have proven particularly informative; by mimicking the layered architecture of the epidermis and dermis, as demonstrated in seminal work generating pluripotent stem-cell-derived skin constructs [97], they create an experimental setting where epithelial regeneration, dermal remodeling, and immune interactions can be examined with unprecedented precision.

Skin organoids offer a unique advantage in fulfilling the call for dynamic analysis through their capacity for autonomous self-organization. Unlike static scaffolds where cell placement is pre-determined, organoids undergo a time-resolved developmental program that mirrors embryonic skin morphogenesis and adult scarless healing. This allows for the study of ‘dynamic’ cellular repositioning and spontaneous niche formation over several weeks. By monitoring organoids during wound-like perturbations, we can observe the temporal sequence of tissue folding, basement membrane maturation, and epidermal stratification in real-time, providing a deeper understanding of the regenerative clock that governs human skin repair.

Recent studies specifically emphasize the value of organoids for modeling core phases of wound healing, such as re-epithelialization, inflammation, and ECM deposition, under conditions that closely approximate in vivo physiology [98]. These systems allow for the detailed observation of keratinocyte migration dynamics, fibroblast-mediated ECM production, and even early vascular responses, all of which are critical for interpreting the pathophysiology of chronic wounds and fibrotic scarring. The integration of immune components into organoid platforms, an area of rapid development in regenerative dermatology, further enhances their predictive power by capturing the inflammatory cues that shape tissue repair outcomes [99].

Moreover, the emergence of patient-derived organoids has opened new avenues for personalized medicine. Because these constructs preserve donor-specific cellular behaviors and molecular signatures, they enable the preclinical testing of biomaterials, growth factors, and cell-based therapies prior to clinical application, aligning with precision-medicine strategies [100]. As bioengineering approaches continue to evolve, particularly through advances that incorporate functional vasculature, resident immune cells, and biomechanical stimuli, organoid platforms are expected to become even more physiologically representative. This trend supports the broader research trajectory outlined in reviews of wound healing and skin regeneration, ultimately positioning organoids as a critical bridge between fundamental laboratory research and translational therapeutic innovation [101,102] (Table 4).

### 8.1. Clinical Success and Regulatory Challenges

Patient-derived organoids have opened new avenues for personalized medicine, allowing for the preclinical testing of cell-based therapies before they reach the patient. They excel in modeling the early inflammatory and re-epithelialization phases of human healing.

Most current organoid systems lack fully integrated innervation and long-term immune competence. Their high variability in morphology and behavior between batches complicates the creation of a standardized regulatory framework for their use in drug development.

### 8.2. Advantages and Limitations

Despite their considerable promise, organoid-based wound-healing models present a combination of notable advantages and inherent limitations that must be carefully considered. Among their strengths, organoids offer a level of structural and functional fidelity far superior to traditional two-dimensional cultures, enabling investigators to study epithelial regeneration, stromal interactions, and inflammatory signaling within a highly biomimetic microenvironment. Indeed, as described in broad reviews of 3D organoid cultures, the shift from 2D to 3D greatly improves physiological relevance and tissue-specific architecture [100].

Their modularity also allows for the targeted manipulation of cellular composition, extracellular matrix (ECM) components, and mechanical cues, thereby facilitating controlled experimentation on specific pathways relevant to tissue repair. Furthermore, patient-derived organoids provide a powerful platform for precision medicine by capturing the inter-individual variability that influences healing capacity and therapeutic responsiveness [103].

However, key limitations remain; most organoid systems still lack fully integrated vasculature, innervation, and long-term immune competence, restricting their ability to fully replicate the dynamic physiological context of in vivo wound repair [104]. Additionally, challenges in standardization, reproducibility, and scalability persist, as organoid cultures can vary significantly in morphology and behavior across laboratories and even between batches [98]. Finally, although advances in bioengineering are progressively addressing these gaps, current models often fall short in predicting late-stage remodeling, scar formation, or systemic influences on healing. This underscores the need to interpret organoid-based findings in conjunction with animal models and clinical data [105] (Figure 2).

A potential solution to these weaknesses could be achieved by integrating organoid technology with organ-on-chip platforms, creating “organoid-on-a-chip” systems. While organ-on-chip models are engineered to mimic the extrinsic characteristics of organs, including dynamic mechanical forces, fluid flow, and biochemical gradients [77], organoids provide a self-organizing, multicellular architecture that faithfully reproduces intrinsic tissue-specific functions [96]. For instance, interactions between neurons and keratinocytes, as well as the formation of vascular structures, are highly dependent on appropriate spatial organization [63]. By combining these two technologies, organoids-on-chips achieve spatial and functional cooperation, serving as highly relevant 3D organotypic models. This enables them to recapitulate complex properties, including cellular heterogeneity, tissue–tissue interfaces, and organ-level functions, while simultaneously allowing for precise manipulation, genetic editing, and high-resolution analysis [77,106,107].

To date, wound-on-a-chip technologies remain in their early stages; nevertheless, their potential is undeniable. Although current models do not yet fully meet the diverse demands of clinical and research applications, advances in bioengineering offer promising opportunities to enhance the structural complexity and functional fidelity of next-generation engineered skin models.

## 9. Stem Cell Technologies

The advent of induced pluripotent stem cells (iPSCs) has introduced a new level of personalization to in vitro wound models previously treated. iPSC-based models can simulate pathological conditions (e.g., diabetes, aging, chronic inflammation) and evaluate cellular responses to biomaterials, growth factors, or gene therapies, bridging toward personalized regenerative medicine [108,109]. By deriving keratinocytes, fibroblasts, and endothelial cells from patient-specific iPSCs, researchers can model individual variability in healing capacity and investigate disease-specific impairments such as diabetic ulcers, autoimmune blistering diseases, or chronic venous wounds [61,110].

Combining iPSC technology with CRISPR/Cas9-mediated gene editing enables the generation of isogenic control lines to dissect the contribution of specific genetic mutations or signaling pathways to impaired wound repair [111]. This approach is particularly valuable in elucidating the molecular basis of rare skin disorders and tailoring patient-specific therapeutic strategies [21,112] (Table 5).

### 9.1. Clinical Success and Regulatory Challenges

Induced pluripotent stem cells (iPSCs) have succeeded in modeling specific disease impairments, such as diabetic ulcers and autoimmune blistering diseases, by using patient-specific cell lines.

Safety remains a paramount concern; the risk of teratoma formation from residual pluripotent cells necessitates rigorous quality control. Furthermore, the lack of standardized guidelines for clinical-grade manufacturing and the epigenetic heterogeneity of iPSC lines remain significant obstacles to their therapeutic application.

### 9.2. Advantages and Limitations

Despite the great promise of iPSC technology for regenerative medicine, several challenges must still be addressed before it can become a reliable tool for therapeutic practice. The generation of iPSCs remains a complex and costly process, requiring advanced expertise, specialized equipment, and expensive reagents; this currently confines its use primarily to experimental protocols and limits scalability for clinical applications [113].

Even after successful reprogramming, directing iPSCs toward fully mature and functional cell types presents additional hurdles, as differentiation protocols vary significantly between laboratories, leading to inconsistencies in cellular functionality and reproducibility [114]. Moreover, iPSC lines exhibit notable epigenetic heterogeneity, where differences in DNA methylation, histone modifications, and chromatin accessibility influence their differentiation potential and behavior, further complicating therapeutic predictability [114,115]. Safety concerns also remain paramount: residual pluripotent cells carry the risk of teratoma formation, and the reprogramming process itself may introduce genomic instability or mutations, necessitating rigorous quality control and innovative strategies to mitigate tumorigenicity [116].

Finally, beyond scientific and technical challenges, regulatory and translational barriers pose significant obstacles, as standardized guidelines for manufacturing, clinical-grade cell production, and quality assurance are still evolving. Addressing these interconnected issues, through the optimization of reprogramming and differentiation protocols, a deeper understanding of epigenetic regulation, and careful safety and regulatory oversight, is essential to fully unlock the clinical potential of iPSC-based therapies [62].

A final summary table (Table 6) relating to the different methodologies covered is provided below.

## 10. Pathological and Disease-Specific Models

While the potential of patient-derived cells in skin modeling is widely acknowledged, a systematic integration of disease-specific adaptations is essential to bridge the gap between general repair models and clinical pathology.

In the context of diabetic wound models, the mere use of patient cells is insufficient without recreating the metabolic microenvironment; this is achieved by incorporating hyperglycemic culture media and the addition of advanced glycation end-products (AGEs), which directly impair fibroblast migration and keratinocyte proliferation [119]. Furthermore, these models must address impaired angiogenesis, often by utilizing endothelial cells pre-conditioned in hypoxic or high-glucose environments to simulate the reduced VEGF signaling characteristic of diabetic stasis [120].

For pressure ulcers, advanced organ-on-chip platforms are employed to replicate ischemia–reperfusion injury, allowing for the precise control of oxygen tension and the application of cyclical mechanical compression to simulate the breakdown of the dermal-epidermal junction [40].

Burn wound models have evolved to include thermal injury simulation through localized heat application on bioprinted constructs, enabling the study of eschar formation and the resulting systemic inflammatory cascade in a controlled 3D architecture [121].

The study of genetic disorders, such as epidermolysis bullosa or psoriasis, has been revolutionized by the use of patient-derived induced pluripotent stem cells (iPSCs) combined with CRISPR/Cas9 gene editing [122]. This approach allows for the creation of isogenic control lines, isolating the specific contribution of genetic mutations to skin fragility or hyperproliferation.

**Figure 4:**
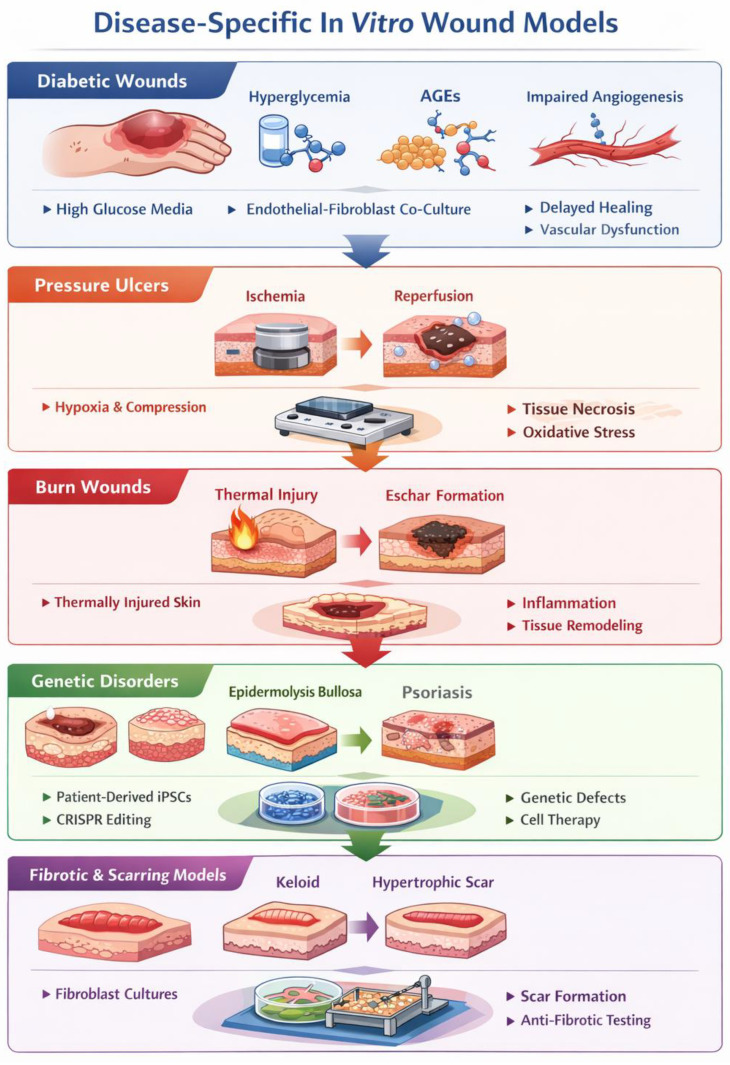
Overview of key in vitro models for wound healing research. The diagram summarizes the etiological factors, culture methodologies (co-cultures, mechanical stress, iPSCs), and primary phenotypic outputs (necrosis, inflammation, fibrosis) associated with various chronic and acute skin conditions (image created by “Google Gemini 3 Flash- free version” AI).

Finally, fibrotic and scarring models, specifically those targeting keloid and hypertrophic scars, leverage high-stiffness synthetic matrices and patient-derived myofibroblasts to study the persistent activation of the TGF-β pathway [117,123] (Figure 4).

Furthermore, these models may serve as pivotal benchmarks for evaluating anti-scarring therapies.

## 11. Current Challenges and Future Perspectives

Emerging technologies and their combination are reshaping in vitro wound healing research. The integration of transcriptomic, proteomic, and metabolomic data, multi-omics, enables a systems-level understanding of tissue repair, moving beyond static analyses of cellular behavior [124,125]. Machine learning approaches further enhance predictive capabilities, supporting the rational design of bioengineered models and, when combined with experimental validation, improving reproducibility and accelerating clinical translation.

Despite these advances, major challenges persist. Limited standardization across laboratories, due to variability in biomaterials, cell sources, 3D printing parameters, and protocols, continues to hinder reproducibility. Moreover, in vitro models still struggle to fully replicate the complexity of wound healing, which involves vascularization, innervation, immune responses, mechanical forces, oxygen gradients, and microbiome interactions [60,117]. Capturing these interdependent processes in a single, scalable model remains both technically and economically demanding.

Nonetheless, emerging technologies are narrowing the gap between experimental models and human physiology. Three-dimensional (3D) bioprinting enables the fabrication of complex tissue architectures with spatially organized cells and biomaterials [22], while microfluidic “wound-on-a-chip” platforms allow for the precise control of mechanical and biochemical stimuli with real-time monitoring [40]. In parallel, iPSC-derived models offer opportunities for personalized approaches to wound healing and targeted therapy testing [62]. Advances in computational tools and in silico modeling further support system design, outcome prediction, and the interpretation of high-dimensional data [118,126].

Looking ahead, the integration of vascular, immune, and microbial components, coupled with real-time biosensing, is expected to enhance the physiological relevance of in vitro wound models. The development of standardized protocols and regulatory frameworks will be essential to ensure reproducibility and clinical applicability. Overall, the convergence of bioengineering, stem cell technologies, microfluidics, and computational modeling is driving the transition toward predictive, human-relevant platforms with significant potential for regenerative medicine, drug development, and translational research.

The transition from static, single-timepoint observations to dynamic, high-resolution temporal modeling is no longer a conceptual preference but a technical necessity. As evidenced by the advancements in microfluidic perfusion and organoid self-assembly, the next generation of in vitro models is moving toward capturing the ‘dynamic wound healing continuum’. By aligning these technologies with the conceptual framework of time-dependent methodologies, researchers can better predict clinical outcomes, moving beyond a simplified ‘healed vs. non-healed’ binary to a comprehensive understanding of the rate and quality of tissue regeneration.

### Integrated Framework for Research and Clinical Applications

The choice of an in vitro model must be guided by the specific phase of wound healing under investigation. For fundamental research into epithelial differentiation, organoids and 3D skin equivalents offer the necessary biological depth. Conversely, for pharmacological screening requiring a “systemic-like” environment, organ-on-chip platforms are superior due to their perfusion capabilities. From a clinical perspective, the convergence of iPSCs and bioprinting holds the most promise for personalized tissue replacement, though regulatory standardization remains the primary hurdle for translation. Ultimately, a “hybrid” approach, such as organoid-on-chip, represents the most comprehensive strategy to bridge the gap between benchtop models and human clinical outcomes.

By addressing these regulatory bottlenecks—primarily through the development of standardized manufacturing protocols and more robust safety profiles—these technologies will be better positioned to achieve full clinical translation and widespread adoption.

## Figures and Tables

**Table 2:** Evolution of Skin Models: Comparison Between Traditional Techniques and 3D Bioprinting in Regenerative Medicine.

Model Type	Key Features	Main Findings	Relevance to Wound Healing
Traditional Engineered Skin [4,23]	Fibroblasts in collagen gel; superficial keratinocyte layer.	Basic epidermal–dermal interaction; monitored matrix remodeling.	Provides a baseline for studying simple re-epithelialization.
Advanced 3D Bioprinted Models [18,20,69,70,71]	Precise spatial deposition; vascular networks; gradients of stiffness.	Mirrors complex topography; enables angiogenesis via VEGF/PDGF.	Enables study of heterogeneous tissue repair and deep-tissue regeneration.
High-Throughput Screening Systems [72,73]	Automated, reproducible fabrication of digital architectures.	Efficient testing of novel biomaterials and pharmacological agents.	Accelerates the discovery of new drugs and bioactive dressings.

**Table 3:** Representative studies on microfluidic wound- and skin-on-chip models relevant to tissue regeneration research.

Model Type	Key Features	Main Findings	Relevance to Wound Healing Research
Microengineered skin model [93]	Human skin equivalent with microvascular networks using endothelial cells and keratinocytes	Demonstrated formation of perfusable vascularized skin constructs	Provides a platform for studying vascularization and tissue regeneration in engineered skin models
Skin-on-a-chip microfluidic system [94]	Microfluidic chip integrating epidermal and dermal layers with controlled perfusion	Reproduced inflammatory responses and edema-like conditions	Enables investigation of inflammatory processes involved in wound healing
Microfluidic wound-healing assay [19]	Microfluidic culture system allowing controlled epithelial injury and monitoring of cell migration	Demonstrated quantitative analysis of cell migration and wound closure dynamics	Useful for studying mechanisms of epithelial repair and screening therapeutic compounds
Full-thickness skin-on-chip model [95]	Multi-layered human skin equivalent with epidermal and dermal compartments	Enabled investigation of tissue regeneration and barrier function under dynamic conditions	Provides physiologically relevant environment to study wound repair and tissue remodeling
Bioelectronic wound-healing model [92]	Platform applying controlled electrical stimulation to wounded epithelial cells	Electrical signals accelerated wound closure and cell migration	Demonstrates the role of bioelectric cues in regulating wound healing processes

**Table 4:** Summary of Skin Organoid Models in Regenerative Research.

Model Type	Key Features	Main Findings	Relevance to Wound Healing
iPSC-Derived Skin Organoids [97]	Pluripotent stem cell-based; autonomous self-organization; mimics embryonic morphogenesis.	Successfully recapitulated layered epidermis, dermis, and even hair follicles.	Provides a “developmental blueprint” for scarless healing and tissue maturation.
Co-culture Systems [96,98]	Integration of keratinocytes and fibroblasts in 3D; layered architecture.	Observed real-time basement membrane maturation and epidermal stratification.	Essential for studying re-epithelialization and dermal remodeling dynamics.
Immune-Integrated Organoids [99]	Inclusion of resident or systemic immune cells (e.g., macrophages/T-cells).	Captures inflammatory signaling pathways and their effect on tissue repair.	Models the inflammatory phase of healing and the pathophysiology of chronic wounds.
Patient-Derived Organoids [100]	Created from donor-specific cells; preserves genetic and molecular signatures.	Maintains donor-specific responses to treatments and biomaterials.	Enables personalized medicine and preclinical testing of targeted therapies.
Vascularized/Biomechanical Models [101,102]	Advanced bioengineering incorporating flow or mechanical tension.	Enhanced physiological representation; improved nutrient transport and stress response.	Bridges the gap between lab research and clinical translation for complex wound care.

**Table 5:** Summary of iPSC-Based Wound Models.

Model Type	Key Features	Main Findings	Relevance to Wound Healing
iPSC-Derived Skin Cells [108,109]	Keratinocytes, fibroblasts, and endothelial cells derived from pluripotent cells.	Ability to simulate pathological conditions like aging and chronic inflammation.	Bridges the gap toward personalized regenerative medicine and biomaterial testing.
Patient-Specific Models [61,110]	Cells derived from patients with specific clinical backgrounds.	Models individual variability and specific disease impairments (e.g., diabetic ulcers).	Investigates the molecular basis of chronic non-healing wounds and autoimmune diseases.
Isogenic Control Lines [111]	Integration of iPSC tech with CRISPR/Cas9 gene editing.	Enables precise dissection of specific genetic mutations vs. environmental factors.	Clarifies the role of signaling pathways in impaired repair processes.
Gene-Edited Therapeutic Models [41,112]	Targeted genetic modification of patient-specific cells.	Elucidates the molecular basis of rare skin disorders.	Facilitates the development of tailored therapeutic strategies for rare conditions.

**Table 6:** Comprehensive Comparison of Wound-Healing Models.

Model Type	Cellular Complexity	ECM Realism	Vascularization	Immune Comp.	Innervation	Dynamic Stimuli	Throughput	Cost
2D Culture [11,37]	Low	Poor	None	None	None	Limited	Very High	Low
Animal Models [70,117]	High	High (Native)	Full	Full	Full	High	Low	High
3D Skin Equivalents [74,76]	Moderate	Good	Limited	Occasional	Rare	Static	Moderate	Moderate
Bioprinting [101,118]	High	High (Bioinks)	Designed	Emerging	Emerging	Moderate	High	High
Organ-on-Chip [40,95]	High	Moderate/High	Perfusion	Integrated	Emerging	High (Shear)	Moderate	High
Organoids [96,97]	Very High	Very High	Spontaneous	Emerging	Emerging	Moderate	Moderate	Moderate
iPSC-derived [108,109,115]	Personalized	Good	High Potential	Emerging	High Potential	Varies	Moderate	High

## Data Availability

No new data was created. The original contributions presented in this study are represented by comments included in the article. Further inquiries can be directed to the corresponding author.
